# *Limosilactobacillus reuteri* as an Adjuvant in the Treatment of Peri-implant Mucositis in Total Rehabilitation: An Exploratory Study

**DOI:** 10.1055/s-0043-1777822

**Published:** 2024-03-31

**Authors:** Gonçalo J.G. Parreira, Miguel A. de Araújo Nobre, André G.C.R. Moreira, Henrique P.S. Luís

**Affiliations:** 1 Universidade de Lisboa, Faculdade de Medicina Dentária, Unidade de Investigação em Ciências Orais e Biomédicas (UICOB), Lisboa, Portugal; 2 Oral Hygiene Department, Maló Clinic, Lisbon, Portugal; 3 Universidade de Lisboa, Faculdade de Medicina Dentária, Rede de Higienistas Orais para o Desenvolvimento da Ciência (RHODes), Lisboa, Portugal; 4 Research and Development Department, Maló Clinic, Lisbon, Portugal; 5 Clínica Universitária de Estomatologia, Faculdade de Medicina, Universidade de Lisboa, Portugal; 6 Center for Innovative Care and Health Technology (ciTechcare), Polytechnic of Leiria, Leiria, Portugal

**Keywords:** probiotic, Limosilactobacillus reuteri, dental implants, peri-implant mucositis, total rehabilitation, mechanical therapy, nonsurgical treatment

## Abstract

**Objectives** Mechanical debridement is the traditional method for the treatment of peri-implant mucositis (P-im) and its success depends on the patient's correct oral hygiene. It is believed that probiotics may help by their ability to modulate the oral biofilm, resulting in anti-inflammatory and antibacterial plaque action. The aim of this study was to evaluate the adjuvant effect of the probiotic *Limosilactobacillus reuteri* (LR) in the mechanical treatment of P-im.

**Materials and Methods** This exploratory study included 29 subjects with implant-supported total rehabilitation and P-im, divided into test (TG) and control (CG) groups, equally subjected to professional mechanical debridement, with the administration of a daily GUM PerioBalance lozenge for 30 days added to the TG. The modified Plaque Index (mPlI) modified Sulcus Bleeding Index (mBI) and pocket depth (PD) were evaluated before the intervention (baseline) and 6 and 10 weeks later.

**Statistical Analysis** Parametric and nonparametric tests with 5% significance level were used in the statistical analysis, using *IBM SPSS Statistics 27.0* software.

**Results** Both treatments resulted in reduced mPlI, mBI, and PD at 6 weeks; while from 6 to 10 weeks there was an increase in mPlI and mBI and maintenance of PD. Compared with baseline, differences were close to statistical significance in the reduction in PD at 10 weeks in the CG ( *p* = 0.018), after Bonferroni correction, and statistically significant in the mPlI at 6 weeks in the CG ( *p* = 0.004) and in the TG ( *p* = 0.002) as well as at 10 weeks in the TG ( *p* = 0.016). Comparing the groups in the postintervention assessments, no statistically significant differences were found.

**Conclusion** LR adjuvant mechanical treatment of P-im does not show a clear benefit compared with mechanical treatment alone, with both interventions achieving similar clinical results. Further prospective and long-term studies are needed.

## Introduction

Dental implants are one of the preferred treatment options for rehabilitating missing teeth. 1 However, the increased use of this alternative has brought about an increase in biomechanical, iatrogenic, aesthetic, and biological complications. 2

Peri-implant mucositis (P-im) presents as inflammation around an implant and is commonly considered a precursor of peri-implantitis. 3 4 According to previous studies, 5 the prevalence of P-im ranges between 43 and 55.6%; while for peri-implant disease, a range between 13.9 and 22% was reported. 5 6 7 Although the considerable prevalence and wide discussion by the scientific community, these complications have no standard therapeutic to be followed. 8 9

While mechanical removal is considered the most effective method of biofilm control, 10 the literature points to the limited effectiveness of nonsurgical professional mechanical debridement (PMD) in the treatment of peri-implant pathologies, regardless of the use of adjunctive treatments. 11 Namely, the use of chemotherapeutic mouth rinses to reduce inflammation and prevent oral diseases by controlling the oral biofilm is common practice, but these are associated with a limitation in the transposition to the periodontal sulcus to less than 2 mm. 12 This restriction is further increased in fixed prosthetic rehabilitations, which are known to be challenging for the patient to clean. Consequently, high levels of bacterial plaque accumulation occur on their surfaces, 13 deeming necessary to explore alternatives and complementary methods.

The Food and Agriculture Organization of the United Nations and the World Health Organization have defined probiotics as live microorganisms that when administered in adequate amounts confer health benefits to the host. 14 They help to re-establish the balance of flora after a process of dysbiosis, particularly in the oral cavity, creating a biofilm that protects the tissues against pathogens by establishing themselves in areas that they would tend to colonize. 15 16 In oral health, various bacterial strains of probiotics have been recognized for their ability to mitigate certain conditions in the oral cavity: prevention of dental caries, by reducing the levels of *Streptococcus mutans* , 17 18 19 treatment of oral candidiasis, 20 21 control of halitosis, 22 23 prevention and treatment of periodontal diseases, 24 25 26 27 as well as peri-implant diseases, 28 29 30 with effects on reducing the amount of bacterial plaque and gingival bleeding justified by reducing the concentrations of cytokines that mediate inflammatory processes. 28 31 32

A systematic review and meta-analysis on the impact of various oral probiotics on chronic periodontitis registered significant improvements for all clinical parameters and pathogens studied, when the administration of oral probiotics was performed as adjuvant to scaling and root planing. 33

*Limosilactobacillus reuteri* (LR) are bacteria capable of reshaping the composition of the commensal microbiota in the host through the synthesis of reuterine (β-hydroxypropionaldehyde), an antimicrobial compound that inhibits the action of gram-negative and positive bacteria, along with other fungi and protozoan infections. 34 An *in vitro* study conducted by Widyarman and Theodorea 35 found promising advantages of this compound against single-species and dual-species periodontal bacterial biofilms.

Thus, it is considered that using prebiotic modulation or LR direct supplementation may constitute attractive preventive/therapeutic measures against peri-implant pathologies. This consideration was based on previous studies reporting a positive correlation between the reduction in LR colonization and the increase in the incidence of inflammatory diseases, 36 as is the case of P-im.

Even though the adjuvant effects of probiotics have been favorably proven in the PMD of gingivitis and periodontitis, their applicability and success in peri-implant tissues remain controversial, making it necessary to investigate this relationship. The aim of this study was to evaluate the adjuvant effect of the probiotic LR in the mechanical treatment of P-im.

## Materials and Methods

### Study Design

A randomized controlled trial, with a two-group parallel design, was considered to evaluate clinically significant differences between performing PMD alone or adjuvated with the supplement GUM PerioBalance (Sunstar Europe S.A., Etoy, Switzerland), on subjects with P-im in All-on-4 concept rehabilitations.

### Study Population

This study was conducted between January and May 2022 in a private rehabilitation center (Malo Clinic, Lisbon, Portugal). The clinical protocol was approved by the local ethics committee (Ethical Committee for Health, authorization no. 001/2021); informed consent was obtained from all participants, having this investigation been conducted according to the principles outlined in the Declaration of Helsinki on experimentation involving human subjects. This clinical trial was registered at www.clinicaltrials.gov with the number NCT05758103. The study population consisted of adult patients with at least one full-arch implant-supported rehabilitation (Nobel Biocare, Gothenburg, Sweden) who were considered eligible during a dental hygiene appointment.

The general health status of the participants and the medication taken were updated, namely the existence of systemic diseases with coagulation impairment likely to compromise the tissue healing process. The patients were also questioned about smoking habits (type and daily amount of tobacco consumed).

### Inclusion and Exclusion Criteria

Inclusion criteria were; (i) patients with dental implants placed for at least 12 months according to the All-on-4 concept; (ii) removal of the dental prosthesis as part of the conventional implant maintenance protocol; (iii) modified Bleeding Index (mBI) 37 score more than 0 in at least one implant in the studied rehabilitation; (iv) implants connected to the prosthesis by means of transepithelial abutments; (v) if there were natural teeth in opposing arch, they were periodontally healthy or had been treated for periodontitis and were on periodontal support with residual pockets less than or equal to 5 mm; (vi) demonstrated previous compliance with oral hygiene appointments; (vii) read and signed the informed consent.

The exclusion criteria were patients who met the following characteristics: (i) Peri-implantitis proven clinically (implant mobility, suppuration and/or pocket depth (PD) ≥5 mm) and/or radiographically (bone remodeling greater than 2 mm in the first year of function 38 and mean marginal bone loss greater than 0.2 mm for each subsequent year 39 in the rehabilitated arch that was intended to be studied); (ii) clinically active peri-implantitis (mobility, suppuration and/or PD ≥5 mm) in the opposing arch to the one intended to be studied; (iii) presence of an extra-maxillary/zygomatic implant in the rehabilitated arch that was intended to be studied; (iv) current probiotic supplementation; (v) diabetes mellitus not controlled by medication; (vi) current use of oral hygiene products containing chlorohexidine or essential oils; (vii) special needs individuals who depended on others for their oral hygiene and medication uptake.

### Randomization

The participants meeting the inclusion criteria and signing the informed consent were selected by convenience at a dental hygiene appointment with the principal investigator. Considering the Consensus Report of the VII European Workshop on Periodontology 40 that determined bleeding on gentle probing (<0.25 Newtons) as the elementary parameter in P-im diagnosis, it was defined that a value greater than score 0 on the mBI 37 and absence of pathological peri-implant bone loss would be associated with a P-im condition. The first selected subject was randomly assigned to test group (TG) or control group (CG) using a toss coin program simulator ( https://flipsimu.com/ ). The participants were alternately distributed until allocating at least 15 participants in each study group, which proceeded simultaneously and independently throughout the research. The principal investigator was responsible for randomization, data collection and analysis.

### Outcome Measures and Clinical Procedures

Primary outcome measure was mBI. 37 Secondary outcome measures were the modified Plaque Index (mPlI) 37 and PD. The intervention started with removing the prosthesis and recording mPlI, mBI, and PD in four spots per implant (mesial, distal, buccal, and palatal/lingual) using the Click-Probe (Kerr-Hawe, Bioggio, Switzerland). Afterward, prophylactic procedures were performed with removal of soft and hard deposits with a polyether-ether-ether-ketone coated tip (Instrument PI; EMS, Nyon, Switzerland), polishing the abutments with a rubber cup (Hawe Prophy Cup; Kerr-Hawe, Bioggio, Switzerland), and 0.2% chlorhexidine gel (PerioKIN; KIN, Barcelona, Spain). The prostheses were decontaminated with a sodium bicarbonate powder jet (AIR-FLOW CLASSIC; EMS, Nyon, Switzerland) and after reconnection, the prosthetic screw access holes were provisionally sealed.

The test group was provided with a pack of the probiotic supplement *Limosilactobacillus reuteri Prodentis* (combining *L. reuteri* DSM 17938 and *L. reuteri* ATCC PTA 5289 strains) (GUM PerioBalance; Sunstar Europe S.A., Etoy, Switzerland), with these participants taking one lozenge daily after night brushing for 30 days and handing in the empty pack at the next evaluation as alleged proof of use. The CG followed without this intervention. No oral hygiene instructions were given to either group, nor participants were requested to modify previously adopted oral hygiene habits during the study. In both groups, besides the assessment of the clinical parameters evaluated at baseline, two more evaluations were performed at 6 and 10 weeks (W). The period stipulated for the second assessment (6W after baseline) was due to the premise that the period of complete resolution of P-im may require periods longer than 3 weeks. 10 In this assessment, the prostheses were removed, and the clinical parameters were assessed, followed by prosthesis reconnection. The third evaluation (10W after baseline) was performed to understand whether the results obtained in the previous evaluation changed.

### Statistical Analysis

Descriptive and inferential statistics were performed on the variables of interest. The unit of analysis was the patient, who had at least one implant with P-im per jaw in the study (single-arch rehabilitations with at least one implant affected by P-im; bimaxillary rehabilitations with at least one implant affected by P-im in each arch). The mean mPlI, mBI, and PD were recorded per time point of assessment.

Normality of data was evaluated with the Kolmogorov–Smirnov test. The repeated measures analysis of variance (ANOVA)/Friedman test was used to evaluate differences within the same group for the mPlI, mBI, and PD over the various assessment times. When significant differences were observed, the Wilcoxon test/paired-samples *t* -test with Bonferroni correction was used to detect the period they occurred. The independent sample *t* -test/Mann–Whitney U test was used to assess potential preintervention differences between the GC and TG for age, time elapsed since surgery and toothbrush frequency variables; and for mPlI, mBI, and PD at baseline, 6 and 10 weeks. The significance level was set at 5%. The data was analyzed using IBM SPSS Statistics 27.0 (SPSS Inc., Chicago, Illinois, United States).

### Power Analysis

The calculation of the study sample size, considering implants with P-im, established 14 participants per group (TG or CG) provided a statistical power of 95% to detect a real clinical difference of 1 mm in PD at implant level between the TG and CG, accepting a significance level of 5%, and a two-sided β of less than 0.05. Additional patients were recruited to account for potential dropout (10%). 41

## Results

### Subjects

**Fig. 1:**
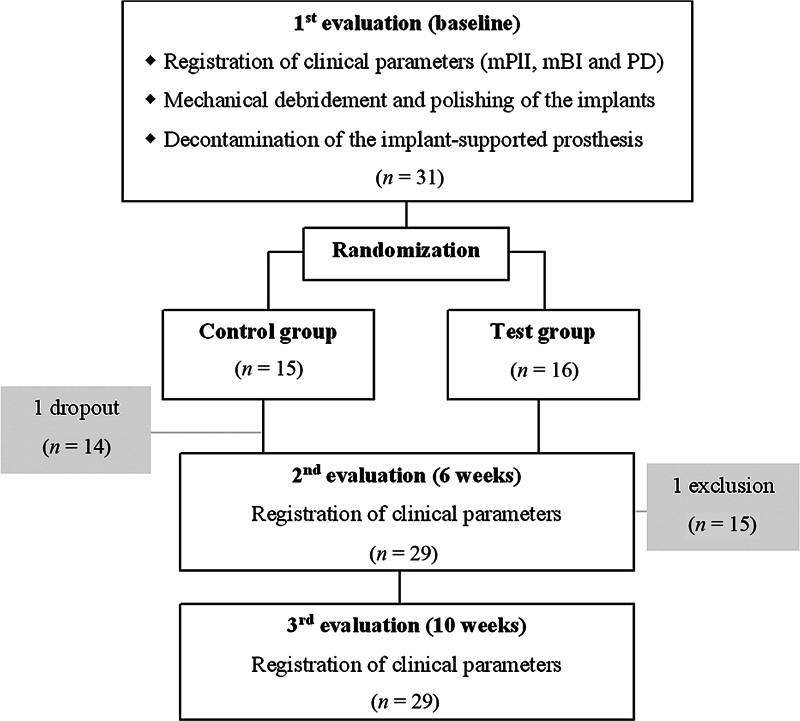
Flowchart of evaluation times and procedures of study groups. mBI, modified Sulcus Bleeding Index; mPlI, modified Plaque Index; PD, pocket depth.

Thirty-one individuals were selected to constitute the sample for this study. There was one dropout in the CG and one member of the TG excluded for missing at 6 weeks. The data collection and interpretation were performed for 29 participants, 14 allocated to CG and 15 to TG ( Fig. 1 ).

### Demographic Parameters

The demographics at baseline are displayed in Table 1 . No significant differences were registered for age, time elapsed since surgery and toothbrush frequency between TG and CG ( *p* >0.05).

**Table 1:** Demographics of the study population at baseline

Variable	Control group	Test group	*p* -Value	Test
Age (years) (mean ± SD)	65.36 ± 9.920	66.13 ± 9.992	0.835	Independent sample *t* -test
Time elapsed since surgery (years) (mean ± SD)	7.36 ± 4.618	7.2 ± 2.908	0.914	Independent sample *t* -test
Toothbrush frequency (mean ± SD)	2 ± 0.842	2 ± 0.828	0.747	Mann–Whitney U test

Abbreviation: SD, standard deviation.

### Health Problems, Medication, and Habits with Potential Impact on Tissue Coagulation and Healing

The most common health problem identified in this sample was cardiovascular disease, with prevalence of 64.3% ( *n* = 9) and 60% ( *n* = 9) in the CG and TG, respectively. About 42.9% ( *n* = 6) of CG and 26.7% ( *n* = 4) of TG took antiplatelet medication. One individual (7.1%) with type 2 diabetes mellitus was allocated to the CG and three (20%) to the TG, all of them medicated with oral antidiabetics. About 21.43% ( *n* = 3) of the CG and 13.23% ( *n* = 2) of the TG participants were smokers. Among these, the daily average was 15 cigarettes (standard deviation [SD] = 4.7256) for the CG and 19 cigarettes (SD = 15.556) for the TG, a nonsignificant difference ( *p* = 0.715; Mann–Whitney U test). None of the smoking participants were identified as taking antiplatelet medication, ruling out a possible antagonistic and overriding action of these two factors on the bleeding response.

### Compliance and Adverse Effects

All TG participants brought the empty pack on the day of the second evaluation (6 weeks). Only one individual reported to have forgotten to take the probiotic twice on nonconsecutive days, while the remaining participants reported having consumed the tablets as per instruction. No adverse effects were reported by the participants or perceived by the investigator in charge.

### Analysis of Clinical Parameters

The results in both groups are depicted in Table 2 . Average mPlI was characterized by the presence of plaque in all evaluations on both groups; bleeding (mBI) was mild on average for both groups; while PD values were around 1 mm.

**Table 2:** Mean values of clinical parameters at the time of evaluation (baseline, 6 and 10 weeks) and difference of means per evaluation period and study group

Clinical parameter	Study group	Moment/period								
		BL (average)	BL-6W (≠average) (%)		6W (average)	6W-10W (≠average) (%)		10W (average)	BL-10W (≠average) (%)	
mPlI	Control	1.84	**↓**	0.35 (19)	1.49	**↑**	0.14 (9.4)	1.63	**↓**	0.21 (11.4)
	Test	1.96	**↓**	0.62 (31.6)	1.34	**↑**	0.21 (15.7)	1.55	**↓**	0.41 (20.9)
mBI	Control	0.79	**↓**	0.03 (3.8)	0.76	**↑**	0.42 (55.3)	1.18	**↑**	0.39 (49.4)
	Test	0.95	**↓**	0.17 (17.9)	0.78	**↑**	0.20 (25.6)	0.98	**↑**	0.03 (3.2)
PD	Control	1.35	**↓**	0.30 (22.2)	1.05	**↑**	0.01 (1)	1.06	**↓**	0.29 (21.5)
	Test	1.18	**↓**	0.26 (22)	0.92	**↓**	0.01 (1.1)	0.91	**↓**	0.27 (22.8)

Abbreviations: ↑—increase; ↓—reduction; 10W, 10 weeks; 6W, 6 weeks; BL, baseline; mBI, modified Bleeding Index; mPlI, modified Plaque Index; PD, pocket depth.

### Evaluation of Clinical Parameters within the Study Groups

#### Control Group

Significant differences were identified between the three assessment times for mPlI ( *p* = 0.026; Friedman test) between BL and 6W ( *p* = 0.004; Wilcoxon test with Bonferroni correction – *p* <0.016), with the mean decreasing by 0.35 (19%; Table 2 ).

The mean mBI showed a decrease of 0.03 (3.8%) in the BL-6W period ( Table 2 ), but after that interval the increase was consistent until the end of the study, ending with an increase of 0.39 (49.4%) in P-im severity compared with the value obtained in BL. There were no significant differences between the three assessments ( *p* = 0.144; Friedman test).

Significant differences were registered between the three assessment periods for the PD ( *p* = 0.038; ANOVA of repeated measures), but when the intervals were evaluated, no significant difference was observed, with the mean increasing by 0.29 (21.5%) during the study period ( Table 2 ).

#### Test Group

Significant differences between the three time points were registered for mPlI ( *p* = 0.001; Friedman test), namely between BL and 6W ( *p* = 0.002; Wilcoxon test with Bonferroni correction) and BL and 10W ( *p* = 0.016; Wilcoxon test with Bonferroni correction), with the mean reduced by 0.62 (31.6%) and 0.41 (20.99%), respectively ( Table 2 ).

No significant differences were registered in the action of the probiotic combined with PMD, both in the clinical parameter mBI ( *p* = 0.207; ANOVA of repeated measures) and PD ( *p* = 0.128; Friedman test).

### Evaluation of Clinical Parameters between Study Groups

No significant differences were registered between CG and TG at any of the assessment periods (baseline, 6 weeks, and 10 weeks) for the clinical parameters studied, mPlI, mBI, and PD ( Table 3 ).

**Table 3:** Summary of the statistical tests used to compare between groups and their statistical significance by clinical parameter evaluated and evaluation time

		Moment		
		Baseline (Sig.)	6 weeks (Sig.)	10 weeks (Sig.)
Clinical parameter	mPlI	0.290 a	0.420 b	1.55 a
	mBI	0.591 a	0.591 a	1.18 b
	PD	0.201 a	0.564 b	0.495 b

a Mann–Whitney U test.

b Independent samples *t* -test.

## Discussion

This study revealed no significant differences between the adjunct use of LR on PMD and PMD alone in the control of P-im. A decrease in all clinical parameters was registered in the BL-6W period for both groups, followed by an increase in the 6W-10W period (except for PD for TG). All clinical parameters did not exceed the baseline values at the final evaluation (10W), except for mBI in both groups. In similar manuscripts to the this investigation, 29 41 42 43 44 a decreasing trend was also seen in all clinical parameters at the assessment following BL in both TG and CG, followed by an increase at the next assessment. Contrary to Galofré et al 41 and Peña et al, 44 there were no significant differences in plaque reduction comparing the final and initial evaluation of PMD alone, and this approach was effective in the very short term. On the other hand, the probiotic combined with PMD appeared to delay biofilm recolonization of the peri-implant region, as in another investigation, 28 where significant plaque reduction occurred in TG given the same probiotic supplementation and dosage as in this investigation. The explanation for the persistence of bleeding in this investigation, even in TG, may be due to the limited ability of PMD in decontaminating the implant surface, 42 aggravated by the implant–abutment connection, which translates into the presence of a micro-gap responsible for harboring a bacterial niche and consequently interfering with peri-implant tissue health. 45 46 This implies that despite a careful attempt to remove deposits from the peri-implant surfaces in BL, which culminated in a reduction of mPlI at the end of the study (significant in TG), the macroscopic interpretation does not invalidate the existence of peri-implant biofilm not observable to the naked eye. Another bacterial niche that may have been present in this study was the cylinder-abutment connection. This interface was disturbed in the 6W evaluation with the removal of the structure to assess the peri-implant clinical parameters. Bacterial plaque may have collapsed into the peri-implant sulcus during placement of the prosthesis, as no prophylaxis of the prosthetic components was performed at this stage. In TG, it was only in this period, 6W to 10W, that no significant differences in mPlI were registered, highlighting the correlation of this parameter with mBI. 47 Some authors argue that the presence of intact biofilm may compromise the effect of probiotics. 42 48 This may justify the fact that although mBI was the only parameter whose mean increased at 10W in both study groups, the TG had a mean score increment of 0.03 compared with 0.39 of the CG. Similarly, Flichy-Fernández et al, 28 in participants diagnosed with P-im in implant-supported full-arch rehabilitations, found a very similar modified gingival index score in subjects submitted to PMD with probiotic support (reduction of 0.09 between BL and final evaluation), compared with a significant increase of almost half a point (0.48) for subjects submitted to PMD alone.

The PD decreased throughout this investigation in both groups (difference of 0.02 mm between them). Despite this, only in the CG were significant differences observed, due to the slightly higher PD at BL. A similar difference, 0.12 mm, was obtained in a systematic review and meta-analysis, 49 but with the particularity being more pronounced and in favor of the TG, although not statistically significant. The PD obtained in this investigation was about half of that reported in a systematic review 50 for PMD alone, where a mean reduction of 0.57 mm was registered. Several authors argued that regardless of the adjuvation of probiotics to mechanical treatment, the mean PD was less than 1 mm, 41 43 44 49 50 which is in line with the results obtained in this study. The baseline mean PD was already low in both groups, and therefore a smaller margin of improvement could be expected over the course of the investigation. Despite PD exhibited a constant downward trend in the TG, no LR potential was attributed to this clinical parameter in the BL-10W period, with the CG assuming a difference very close to statistical significance in the same period, even with a very residual upward trend between 6W and 10W (0.01 mm). Furthermore, a meta-analysis of seven randomized controlled trials 51 evaluating the effect of LR on PD concluded that although there was a slight reduction in PD associated with LR intake, its effect was limited.

The study groups in this investigation were analogous for clinical parameters assessed at BL, with slightly higher values in mPlI and mBI for TG and PD for CG. However, as there were no significant differences between groups in this preintervention phase, it can be inferred that they did not influence the results obtained after treatment. These minor differences between groups may be related to the small sample size, as Galofré et al 41 in a clinical trial with a sample of 22 participants diagnosed with P-im also found a slightly higher percentage of plaque in the TG, also not significant ( *p* = 0.330). In addition, the same situation occurred in the clinical trial by Mongardini et al, 42 which included 20 implant-supported crowns from 20 participants with induced P-im, where a slightly higher level of bleeding on probing in the TG and of mPlI in the CG was obtained prior to treatment, also without significant differences ( *p* > 0.05). In the clinical trials conducted by Galofré et al 41 and Peña et al, 44 in which 1 tablet per day of LR was administered for 30 days to TG participants, no significant differences were found between the study groups at any of the postintervention assessment time points for plaque and peri-implant bleeding on probing percentages as well as for PD. Hallström et al 43 applied a reinforced dosage and duration of probiotic therapy (two daily doses of the same probiotic for 3 months), and similarly, found no significant differences between study groups at 4, 12, and 26 weeks. In agreement with the aforementioned articles, no significant differences were found in this investigation for the clinical parameters mPlI, mBI, and PD between the study groups at any of the assessments after the intervention. Therefore, a supplementary adjuvant effect of LR to PMD cannot be attributed.

Several clinical trials have proven the efficacy of LR as an adjunct to scaling and root planning in controlling moderate-to-severe chronic periodontitis, obtaining significant differences compared with the mechanical approach alone for all clinical parameters evaluated (plaque index, gingival index, bleeding on probing, PD and clinical attachment level). 24 27 52 These results were similar whether a full mouth treatment had been performed beforehand 24 as in this study, or a 1-week interval was applied between two sessions. 27 52 The differences between the cited references and this study might be explained by sample demographic characteristics and methodology: The cited studies involved participants with considerably lower age 24 27 52 compared with the sample of this study, where the age of the participants allocated them to a senior age group, with a tendency to begin neglecting self-hygiene and oral care. 53

Furthermore, the sex distribution rendered a higher proportion of females in this study, whereas a balanced allocation 24 27 or higher proportion of males 52 was found in the cited references. In general, women tend to be more proactive and positive about dental visits compared with men, potentially justifying part of the differences. 54 55

Concerning the differences in methodology, the number and timing of lozenges taken in these studies (two per day after morning and evening brushing for 3 weeks) 27 52 represented a higher dose but a shorter treatment regimen (1 week less) compared with this study. This increase in dosage may have been justified by the fact that periodontitis is a more complex gingival pathology to control than P-im, although the latter has a better prognosis when detected and managed early, since it can easily develop a more severe peri-implant pathological entity, with bone resorption from microbiological origin. This is because there are structural differences between the tooth and the implant, as well as the tissues that surround them. Unlike the tooth, the implant has no periodontal ligament and a weaker mucosal seal, liable to harbor a bacterial infiltrate that triggers a higher proinflammatory state when compared with periodontal tissues. 56 In addition, the fact that the implants studied served as a support for an implant-supported prosthesis, which is a unique and large structure that is difficult to sanitize, may also have compromised the effectiveness of the daily oral hygiene performed by the participants. 13 The results obtained may have been influenced by some limitations present in this investigation: (i) due to the principal investigator being working alone in the field, there was no blinding of the examiner or the participants, both being aware of the study group to which they were allocated; (ii) data were interpreted based on the confidence of cooperation and compliance with the reported probiotic intake and delivery of the empty container by the TG individuals; (iii) the interpretation of numerous practical and theoretical concepts of P-im as well as the diversity of criteria and gingival indices used in the diagnosis, extent and severity of this condition in the literature, may potentially influence the diagnosis of this condition making it difficult to compare with other studies; (iv) the mean age of the participants in this study was considerably higher than other studies on this topic, as most studies addressed P-im in single or partial rehabilitations, associated with younger populations; (v) due to the vasoconstrictor action of nicotine, the inclusion of smokers, although few and well distributed among the study groups, may have contributed to reduce the severity of mBI. The strengths of this investigation include the study design (randomized controlled trial) that accounts as a benchmark for studying causal relationships between interventions and outcomes as randomization eliminates part of the bias inherent with other study designs. 57 An additional strength of this investigation was performing the study in a theme where the literature is very scarce.

Further prospective studies are needed, with systematized variables and conducted in the long term.

The practical implications of the findings relate to the outcome on the short term. This study found no significant benefit of probiotic support as adjuvant to PMD. Clinicians should be aware that the main lead in establishing conditions to revert the effects of P-im remains the mechanical action obtained during proper self-care by the patient as previously described in the literature. 10

## Conclusion

No significant differences were found in peri-implant clinical parameters at any of the postintervention assessments when comparing PMD alone with PMD with probiotic support. When comparing the final evaluation with the preintervention evaluation, the probiotic allied to the PMD contributed to a significant reduction in plaque, although without relevant clinical gains. During this period, the group receiving PMD alone achieved a reduction close to statistical significance in the probing depth, but also without clinical significance. Considering the methodology used and within the limitations of this study, no clinical benefit in P-im was attributed for the adjuvant action of LR on PMD compared with PMD alone.

## References

[1] Alam M K Rahman S A Basri R Sing Yi T T Si-Jie J W Saha S Dental implants - perceiving patients' satisfaction in relation to clinical and electromyography study on implant patients PLoS One 2015 10 10 e0140438 26465146 10.1371/journal.pone.0140438 PMC4605640

[2] Jung R E Zembic A Pjetursson B E Zwahlen M Thoma D S Systematic review of the survival rate and the incidence of biological, technical, and aesthetic complications of single crowns on implants reported in longitudinal studies with a mean follow-up of 5 years Clin Oral Implants Res 2012 23 06 2 21 23062124 10.1111/j.1600-0501.2012.02547.x

[3] Lang N P Wilson T G Corbet E F Biological complications with dental implants: their prevention, diagnosis and treatment Clin Oral Implants Res 2000 11 01 146 155 11168263 10.1034/j.1600-0501.2000.011s1146.x

[4] Belibasakis G N Microbiological and immuno-pathological aspects of peri-implant diseases Arch Oral Biol 2014 59 01 66 72 24209597 10.1016/j.archoralbio.2013.09.013

[5] de Araújo Nobre M Maló P Prevalence of periodontitis, dental caries, and peri-implant pathology and their relation with systemic status and smoking habits: Results of an open-cohort study with 22009 patients in a private rehabilitation center J Dent 2017 67 36 42 28750777 10.1016/j.jdent.2017.07.013

[6] Salvi G E Cosgarea R Sculean A Prevalence of periimplant diseases Implant Dent 2019 28 02 100 102 30762625 10.1097/ID.0000000000000872

[7] Derks J Tomasi C Peri-implant health and disease. A systematic review of current epidemiology J Clin Periodontol 2015 42 16 S158 S171 25495683 10.1111/jcpe.12334

[8] Claffey N Clarke E Polyzois I Renvert S Surgical treatment of peri-implantitis J Clin Periodontol 2008 35 08 316 332 18724859 10.1111/j.1600-051X.2008.01277.x

[9] Renvert S Roos-Jansåker A M Claffey N Non-surgical treatment of peri-implant mucositis and peri-implantitis: a literature review J Clin Periodontol 2008 35 08 305 315 10.1111/j.1600-051X.2008.01276.x 18724858

[10] Salvi G E Aglietta M Eick S Sculean A Lang N P Ramseier C A Reversibility of experimental peri-implant mucositis compared with experimental gingivitis in humans Clin Oral Implants Res 2012 23 02 182 190 21806683 10.1111/j.1600-0501.2011.02220.x

[11] Schwarz F Jepsen S Herten M Sager M Rothamel D Becker J Influence of different treatment approaches on non-submerged and submerged healing of ligature induced peri-implantitis lesions: an experimental study in dogs J Clin Periodontol 2006 33 08 584 595 16899102 10.1111/j.1600-051X.2006.00956.x

[12] Wunderlich R C Singelton M O'Brien W J Caffesse R G Subgingival penetration of an applied solution Int J Periodontics Restorative Dent 1984 4 05 64 71 6596297

[13] Abi Nader S Eimar H Momani M Shang K Daniel N G Tamimi F Plaque accumulation beneath maxillary all-on-4™ implant-supported prostheses Clin Implant Dent Relat Res 2015 17 05 932 937 24461161 10.1111/cid.12199

[14] Nutrition Division.Probiotics in food: Health and nutritional properties and guidelines for evaluationFood and Agriculture Organization of the United Nations. Published2006. Accessed November 24, 2023 at:https://www.fao.org/documents/card/en/c/7c102d95-2fd5-5b22-8faf-f0b2e68dfbb6/

[15] Comelli E M Guggenheim B Stingele F Neeser J R Selection of dairy bacterial strains as probiotics for oral health Eur J Oral Sci 2002 110 03 218 224 12120707 10.1034/j.1600-0447.2002.21216.x

[16] Çaglar E Kargul B Tanboga I Bacteriotherapy and probiotics' role on oral health Oral Dis 2005 11 03 131 137 15888102 10.1111/j.1601-0825.2005.01109.x

[17] Selvaraj K Bharath N Natarajan R Dinesh S Murugesan S Selvaraj S Comparative evaluation of antimicrobial efficacy of toothpastes containing probiotic and neem as primary ingredient on salivary *Streptococcus mutans* in Melmaruvathur population: an *in vivo* study J Pharm Bioallied Sci 2020 12 01 S595 S600 33149527 10.4103/jpbs.JPBS_209_20 PMC7595475

[18] Cannon M L Vorachek A Le C White K Retrospective review of oral probiotic therapy J Clin Pediatr Dent 2019 43 06 367 371 31657987 10.17796/1053-4625-43.6.1

[19] Çaglar E Cildir S K Ergeneli S Sandalli N Twetman S Salivary mutans streptococci and lactobacilli levels after ingestion of the probiotic bacterium *Lactobacillus reuteri* ATCC 55730 by straws or tablets Acta Odontol Scand 2006 64 05 314 318 16945898 10.1080/00016350600801709

[20] Mendonça F HBP Santos S S Faria IdaS Gonçalves e Silva C R Jorge A OC Leão M VP Effects of probiotic bacteria on Candida presence and IgA anti-Candida in the oral cavity of elderly Braz Dent J 2012 23 05 534 538 23306230 10.1590/s0103-64402012000500011

[21] Li D Li Q Liu C Efficacy and safety of probiotics in the treatment of Candida-associated stomatitis Mycoses 2014 57 03 141 146 23952962 10.1111/myc.12116

[22] Lee D S Lee S A Kim M Nam S H Kang M S Reduction of Halitosis by a tablet containing *Weissella cibaria* CMU: a randomized, double-blind, placebo-controlled study J Med Food 2020 23 06 649 657 32379992 10.1089/jmf.2019.4603

[23] Yoo J I Shin I S Jeon J G Yang Y M Kim J G Lee D W The effect of probiotics on halitosis: a systematic review and meta-analysis Probiotics Antimicrob Proteins 2019 11 01 150 157 29168154 10.1007/s12602-017-9351-1

[24] Teughels W Durukan A Ozcelik O Pauwels M Quirynen M Haytac M C Clinical and microbiological effects of Lactobacillus reuteri probiotics in the treatment of chronic periodontitis: a randomized placebo-controlled study J Clin Periodontol 2013 40 11 1025 1035 24164569 10.1111/jcpe.12155 PMC3908359

[25] Ng E Tay J RH Saffari S E Lim L P Chung K M Ong M MA Adjunctive probiotics after periodontal debridement versus placebo: a systematic review and meta-analysis Acta Odontol Scand 2022 80 02 81 90 34197264 10.1080/00016357.2021.1942193

[26] Alshareef A Attia A Almalki M Effectiveness of probiotic lozenges in periodontal management of chronic periodontitis patients: clinical and immunological study Eur J Dent 2020 14 02 281 287 32438428 10.1055/s-0040-1709924 PMC7274828

[27] Tekce M Ince G Gursoy H Clinical and microbiological effects of probiotic lozenges in the treatment of chronic periodontitis: a 1-year follow-up study J Clin Periodontol 2015 42 04 363 372 25728888 10.1111/jcpe.12387

[28] Flichy-Fernández A J Ata-Ali J Alegre-Domingo T The effect of orally administered probiotic Lactobacillus reuteri-containing tablets in peri-implant mucositis: a double-blind randomized controlled trial J Periodontal Res 2015 50 06 775 785 25712760 10.1111/jre.12264

[29] Alqahtani F Alqahtani M Shafqat S S Akram Z Al-Kheraif A A Javed F Efficacy of mechanical debridement with adjunctive probiotic therapy in the treatment of peri-implant mucositis in cigarette-smokers and never-smokers Clin Implant Dent Relat Res 2019 21 04 734 740 31094086 10.1111/cid.12795

[30] Arbildo-Vega H I Panda S Bal A Clinical effectiveness of *Lactobacillus reuteri* in the treatment of peri-implant diseases: a systematic review and meta-analysis J Biol Regul Homeost Agents 2021 35(2, Suppl. 1):79 88 34281304 10.23812/21-2supp1-7

[31] Twetman S Derawi B Keller M Ekstrand K Yucel-Lindberg T Stecksén-Blicks C Short-term effect of chewing gums containing probiotic Lactobacillus reuteri on the levels of inflammatory mediators in gingival crevicular fluid Acta Odontol Scand 2009 67 01 19 24 18985460 10.1080/00016350802516170

[32] Krasse P Carlsson B Dahl C Paulsson A Nilsson A Sinkiewicz G Decreased gum bleeding and reduced gingivitis by the probiotic Lactobacillus reuteri Swed Dent J 2006 30 02 55 60 16878680

[33] Li J Zhao G Zhang H M Zhu F F Probiotic adjuvant treatment in combination with scaling and root planning in chronic periodontitis: a systematic review and meta-analysis Benef Microbes 2023 14 02 95 107 36856123 10.3920/BM2022.0056

[34] Axelsson L T Chung T C Dobrogosz W J Lindgren S E Production of a broad spectrum antimicrobial substance by lactobacillus reuteri Microb Ecol Health Dis 1989 2 131 136

[35] Widyarman A S Theodorea C F Novel indigenous probiotic Lactobacillus reuteri strain produces anti-biofilm Reuterin against pathogenic periodontal bacteria Eur J Dent 2022 16 01 96 101 34303315 10.1055/s-0041-1731591 PMC8890917

[36] Mu Q Tavella V J Luo X M Role of *Lactobacillus reuteri* in human health and diseases Front Microbiol 2018 9 757 29725324 10.3389/fmicb.2018.00757 PMC5917019

[37] Mombelli A van Oosten M AC Schürch E Jr Land N P The microbiota associated with successful or failing osseointegrated titanium implants Oral Microbiol Immunol 1987 2 04 145 151 3507627 10.1111/j.1399-302x.1987.tb00298.x

[38] Maló P Rangert B Dvärsäter L Immediate function of Brånemark implants in the esthetic zone: a retrospective clinical study with 6 months to 4 years of follow-up Clin Implant Dent Relat Res 2000 2 03 138 146 11359258 10.1111/j.1708-8208.2000.tb00004.x

[39] Albrektsson T Zarb G Worthington P Eriksson A R The long-term efficacy of currently used dental implants: a review and proposed criteria of success Int J Oral Maxillofac Implants 1986 1 01 11 25 3527955

[40] Lang N P Berglundh T Periimplant diseases: Where are we now? - Consensus of the Seventh European Workshop on Periodontology J Clin Periodontol 2011 38(suppl 11):S178 S181 10.1111/j.1600-051X.2010.01674.x 21323713

[41] Galofré M Palao D Vicario M Nart J Violant D Clinical and microbiological evaluation of the effect of Lactobacillus reuteri in the treatment of mucositis and peri-implantitis: a triple-blind randomized clinical trial J Periodontal Res 2018 53 03 378 390 29352461 10.1111/jre.12523

[42] Mongardini C Pilloni A Farina R Di Tanna G Zeza B Adjunctive efficacy of probiotics in the treatment of experimental peri-implant mucositis with mechanical and photodynamic therapy: a randomized, cross-over clinical trial J Clin Periodontol 2017 44 04 410 417 28032908 10.1111/jcpe.12689

[43] Hallström H Lindgren S Widén C Renvert S Twetman S Probiotic supplements and debridement of peri-implant mucositis: a randomized controlled trial Acta Odontol Scand 2016 74 01 60 66 25953193 10.3109/00016357.2015.1040065

[44] Peña M Barallat L Vilarrasa J Vicario M Violant D Nart J Evaluation of the effect of probiotics in the treatment of peri-implant mucositis: a triple-blind randomized clinical trial Clin Oral Investig 2019 23 04 1673 1683 10.1007/s00784-018-2578-8 30151705

[45] Candotto V Gabrione F Oberti L Lento D Severino M The role of implant-abutment connection in preventing bacterial leakage: a review J Biol Regul Homeost Agents 2019 33(3, Suppl. 1):129 134 31538459

[46] Lauritano D Moreo G Lucchese A Viganoni C Limongelli L Carinci F The impact of implant-abutment connection on clinical outcomes and microbial colonization: a narrative review Materials (Basel) 2020 13 05 1131 32138368 10.3390/ma13051131 PMC7085009

[47] Pontoriero R Tonelli M P Carnevale G Mombelli A Nyman S R Lang N P Experimentally induced peri-implant mucositis. A clinical study in humans Clin Oral Implants Res 1994 5 04 254 259 7640340 10.1034/j.1600-0501.1994.050409.x

[48] Teughels W Loozen G Quirynen M Do probiotics offer opportunities to manipulate the periodontal oral microbiota? J Clin Periodontol 2011 38 11 159 177 21323712 10.1111/j.1600-051X.2010.01665.x

[49] Zhao R Hu H Wang Y Lai W Jian F Efficacy of probiotics as adjunctive therapy to nonsurgical treatment of peri-implant mucositis: a systematic review and meta-analysis Front Pharmacol 2021 11 541752 33536901 10.3389/fphar.2020.541752 PMC7847846

[50] Barootchi S Ravidà A Tavelli L Wang H L Nonsurgical treatment for peri-implant mucositis: a systematic review and meta-analysis Int J Oral Implantol (New Malden) 2020 13 02 123 139 32424380

[51] Gao J Yu S Zhu X Yan Y Zhang Y Pei D Does probiotic lactobacillus have an adjunctive effect in the nonsurgical treatment of peri-implant diseases? a systematic review and meta-analysis J Evid Based Dent Pract 2020 20 01 101398 32381407 10.1016/j.jebdp.2020.101398

[52] İnce G Gürsoy H İpçi ŞD Cakar G Emekli-Alturfan E Yılmaz S Clinical and biochemical evaluation of lozenges containing *Lactobacillus reuteri* as an adjunct to non-surgical periodontal therapy in chronic periodontitis J Periodontol 2015 86 06 746 754 25741580 10.1902/jop.2015.140612

[53] Skorupka W Zurek K Kokot T Assessment of oral hygiene in adults Cent Eur J Public Health 2012 20 03 233 236 23285527 10.21101/cejph.a3712

[54] Su S Lipsky M S Licari F W Hung M Comparing oral health behaviours of men and women in the United States J Dent 2022 122 104157 35545161 10.1016/j.jdent.2022.104157

[55] Lipsky M S Su S Crespo C J Hung M Men and oral health: a review of sex and gender differences Am J Men Health 2021 15 03 15579883211016361 10.1177/15579883211016361 PMC8127762 33993787

[56] Emecen-Huja P Eubank T D Shapiro V Yildiz V Tatakis D N Leblebicioglu B Peri-implant versus periodontal wound healing J Clin Periodontol 2013 40 08 816 824 23772674 10.1111/jcpe.12127 PMC3725126

[57] Hariton E Locascio J J Randomised controlled trials - the gold standard for effectiveness research: study design: randomised controlled trials BJOG 2018 125 13 1716 29916205 10.1111/1471-0528.15199 PMC6235704

